# Integrating Significant SNPs Identified by GWAS for Genomic Prediction of the Number of Ribs and Carcass Length in Suhuai Pigs

**DOI:** 10.3390/ani15030412

**Published:** 2025-02-02

**Authors:** Kaiyue Liu, Yanzhen Yin, Binbin Wang, Chenxi Liu, Wuduo Zhou, Peipei Niu, Ruihua Huang, Pinghua Li, Qingbo Zhao

**Affiliations:** 1 Key Laboratory in Nanjing for Evaluation and Utilization of Pigs Resources, Ministry of Agriculture and Rural Areas of China, Institute of Swine Science, Nanjing Agricultural University, Nanjing 210095, China; liukaiyue99@njau.edu.cn (K.L.); 2019105013@njau.edu.cn (Y.Y.); 2018205003@njau.edu.cn (B.W.); 2020205005@stu.njau.edu.cn (C.L.); zhouwuduo@njau.edu.cn (W.Z.); rhhuang@njau.edu.cn (R.H.); 2 Huaian Academy, Nanjing Agricultural University, Huai’an 223005, China; niupeipei2@126.com

**Keywords:** genomic selection, carcass traits, preselected variants

## Abstract

The number of ribs (NRs) and the carcass length (CL) are important economic traits. The traits are usually measured after slaughter. To improve the prediction performance of genomic selection (GS) for NRs and CL, one strategy is to integrate the significant loci identified from whole-genome sequencing (WGS) data by genome-wide association study (GWAS) into the genomic prediction (GP) model. This study investigated the GP of different genomic best linear unbiased prediction (GBLUP) and Bayesian models using chip genotype data, imputed WGS (iWGS) data and modeling significant single-nucleotide polymorphisms (SNPs) in different ways for the GP of NRs and CL in the Suhuai pig population. The prediction accuracy, bias and running time of 15 different GP models were evaluated by 10-fold cross-validation. The prediction accuracy of GBLUP using chip data for NRs and CL was 0.314 ± 0.022 and 0.194 ± 0.040, respectively. For NRs, based on the iWGS data, treating the most significant SNP as fixed effects in the GBLUP model had the highest predictive performance, with a prediction accuracy of 0.528 ± 0.023. For CL, based on the chip data, the model that added all the significant SNPs identified by imputed data by GWAS into the multi-trait GBLUP as the second random additive effect was the highest predictive performance, with a prediction accuracy of 0.305 ± 0.027. This study provides insights into optimizing GP models for small populations with phenotypes that are difficult to measure.

## Simple Summary

Integrating the significant loci identified from genome-wide association study (GWAS) is a strategy to improve the prediction performance of genomic selection. In this study, the significant loci identified by GWAS for carcass traits were integrated into genomic best linear unbiased prediction (GBLUP) and Bayesian genomic prediction (GP) models in different forms. The prediction accuracy, bias and running time of 15 different GP models for the number of ribs and carcass length were evaluated by 10-fold cross-validation to obtain the optimal GP model.

## 1. Introduction

In the Chinese market, pork ribs are very popular among consumers. Pigs with a higher number of ribs (NRs) and longer carcass length (CL) have a higher meat yield [1,2]. Therefore, there is potential to enhance economic values for the genetic improvement of these traits. However, NRs and CL traits are usually measured after slaughter, which makes it difficult for traditional selection to improve the genetic gain of NRs and CL.

Marker-assisted selection (MAS) can be used for these two traits by using the causal genes or mutations that have been identified to affect NRs and CL in pigs. A few crucial genes for NRs or CL have been identified in several quantitative trait loci (QTL), such as the *VRTN* (Vertnin) gene, which is associated with NRs and CL [3,4,5], as well as the *NR6A1* (nuclear receptor subfamily 6 group A member 1) gene [6] and *BMP2* (bone morphogenetic protein 2 gene), which are associated with body length and CL [7]. However, these identified causal genes or mutations cannot explain all the genetic variation. There are still other genes affecting NRs and CL in the pig genome that need to be explored. Thus, the MAS has limited ability to enhance the genetic progress of NRs and CL. The genomic selection (GS) does not need to detect significant associations between genetic markers and traits [8]. By utilizing the genotypic and phenotypic data of a reference population, a prediction equation can be established to estimate the breeding value of selection candidates through their individual genotypes so that the breeding individuals can be selected at an early stage.

The parametric models of genomic prediction (GP) include the best linear unbiased prediction (BLUP) alphabet and Bayesian alphabet methods. The widely used BLUP alphabet methods utilize the relationship matrix among individuals constructed from pedigree or genetic markers to estimate breeding values or genomic breeding values (GEBV) of individuals. The Bayesian alphabet methods first estimate the effect of each genetic marker and then accumulate the individual GEBV based on the genotypes of the individual. The simplest method for GP is the GBLUP model, which assumes that the weights of all SNPs are the same in the construction of the kinship matrix. However, some genetic markers are in linkage disequilibrium (LD) with QTL with large effects, while others are not. Therefore, the assumptions of the GBLUP models may not be appropriate.

To solve this problem, many improved models have been proposed based on the genomic BLUP (GBLUP) model. For instance, the trait-specific relationship matrix BLUP (TABLUP) model assigned different weights to genetic markers [9]; the genomic feature BLUP (GFBLUP) model considered functional mutation known to affect traits as a second random additive effect [10,11] and the multiple random effects BLUP (MultiBLUP) [12] and the marker-assisted BLUP (MABLUP) [13] models used the information from GWAS.

In the Bayesian alphabet method, the BayesA model assumes the effects of different markers have different variances [8]; the BayesB model assumes that a proportion of markers have zero effects and the rest of markers have effects with different variances [8]; the BayesBpi model, based on BayesB, allows the proportion of effective markers to be estimated [14]; the BayesC model assumes that a proportion of markers have zero effects and the rest of markers have effects with same variances [15]; the BayesCpi model, based on BayesC, allows the proportion of effective markers to be estimated [16]; the BayesR model assumes the distribution of marker effects is a mixture of many normal distributions [17], and so on. Previous studies have shown that the prediction accuracy of these Bayesian models is significantly higher than that of GBLUP models when analyzing simulation data but only slightly better than or similar to GBLUP when analyzing real livestock data, dependent on traits.

In the case of indigenous pig breeds with a small population, obtaining a reference population with lots of NRs and high CL is challenging. Consequently, optimizing models for improving the accuracy of GS becomes indispensable. Additionally, there was a genetic correlation between NRs and CL. The multi-trait GBLUP model can utilize information from genetic correlations between traits to improve the predictive ability of genomic selection, compared to the single-trait GBLUP model [18,19]. The aim of this study is to explore the optimal GP model for genetics improvement of NRs and CL in the Suhuai pig population using chip genotype data and iWGS data.

## 2. Material and Methods

### 2.1. Animal Population and Data Processing

In this study, the data were collected from 513 Suhuai pigs [20], which had chip SNP genotypes and both NRs and CL phenotypes. The detailed information of the phenotype was presented in a previous study [20]. The pigs were slaughtered in five batches. To correct the influence of the different types of carcass weight (CW) records, the phenotype data of CL were adjusted for sex, slaughter batch and CW using the lm() function of R software (version 4.1.2) in the first to fourth slaughter batches and the fifth slaughter batch, respectively.

The re-sequencing data were used as a reference panel for genotype imputation, including 1602 pigs of multiple breeds from the PigGTEx project [21] and 60 Suhuai pigs form the previous study [20]. Only the SNPs that were common in the above two sources of reference data were retained, resulting in 17,833,675 SNPs. The Beagle (version 5.2) program with the default parameters was used for the imputation of the SNP-chip dataset to the whole-genome density [22]. The SNPs with a dosage R-squared (DR^2^, which is the accuracy of imputation estimated by Beagle) below 0.9 and MAF less than 0.05 were filtered. After quality control, 11,063,641 SNPs were retained for further analysis.

### 2.2. Significant SNPs Identified by GWAS

To improve the ability of GP for NRs and CL, the significant SNPs associated with traits were identified by GWAS. The GWAS was performed by LDAK (version 5.2) software [23] using the “leave-one-chromosome-out” method [24]. The mixed linear model for GWAS is given by Equation [20]:

y=Xa+Wb+k+e

where y represents the vector of phenotype, a is the vector of fixed effects (for NRs, fixed effects include sex, slaughter batch and the top two principal components; for CL, fixed effects include the different types of CW record and the top two principal components.). ***b*** represents the substitution effect of the alleles, k is the random polygenic effect following ***N***(0, ***K***$\sigma_{A}^{2}$) distribution, ***K*** is the genomic kinship matrix calculated with the LDAK-Thin algorithm under the default parameters, e is the vector of random residual effects following ***N***(0, ***I***$\sigma_{e}^{2}$), $\sigma_{A}^{2}$ is the additive genetic variance, $\sigma_{e}^{2}$ is the residual variance and X and ***W*** are the incidence matrices.

The significance threshold of GWAS was set as 1/*N*. For chip data, *N* was the number of SNPs after quality control. For imputation data, *N* was the number of independent SNPs estimated by PLINK (version 1.9) software [25] with the “--indep-pairwise 50 10 0.5” parameter. The significant SNP was defined as the SNP with a *p*-value less than 1/*N*. The most significant SNP was defined as the SNP both with the lowest *p*-value and reaching the suggestive threshold.

### 2.3. GP Models of BLUP Alphabet Method

ST-GBLUP (single-trait GBLUP):

y=Xa+Zg+e

where y is the vector of observations, ***a*** is the vector of fixed effects (for NRs, fixed effects include sex and slaughter batch; for CL, fixed effects include the different types of the carcass weight records), g is the vector of random polygenic effect, e is the vector of random residual effects and X and Z are incidence matrices. It is assumed that g~*N* (0, ***G***$\sigma_{A}^{2}$) and e~*N* (0, ***I***$\sigma_{e}^{2}$), where ***G*** is the matrix of additive genetic relationships constructed based on chip or iWGS data, ***I*** is the identity matrix, $\sigma_{A}^{2}$ is the additive genetic variance and $\sigma_{e}^{2}$ is the residual variance. The GEBV was defined as $Z\hat{g}$.

MT-GBLUP (multi-trait GBLUP):

$$\left[\begin{array}{c} y_{1}\\ y_{2} \end{array}\right]=\left[\begin{array}{cc} X_{1} & 0\\ 0 & X_{2} \end{array}\right]\left[\begin{array}{c} a_{1}\\ a_{2} \end{array}\right]+\left[\begin{array}{cc} Z_{1} & 0\\ 0 & Z_{2} \end{array}\right]\left[\begin{array}{c} g_{1}\\ g_{2} \end{array}\right]+\left[\begin{array}{c} e_{1}\\ e_{2} \end{array}\right]$$

where $y_{1}$ and $y_{2}$ are the vector of observations for NRs and CL; ***a*_1_** and $a_{2}$ are the vectors of fixed effects for NRs and CL; $g_{1}$ and $g_{2}$ are the vectors of random polygenic effects for NRs and CL; $e_{1}$ and ***e*_2_** are the vectors of random residual effects for NRs and CL, respectively, and $X_{1}$, $X_{2}$, $Z_{1}$ and $Z_{2}$ are incidence matrices. It is assumed that $\left[\begin{array}{c} g_{1}\\ g_{2} \end{array}\right]\;\sim \;N\left(0,G⊗\left[\begin{array}{cc} \sigma_{A1}^{2} & \sigma_{A1A2}\\ \sigma_{A1A2} & \sigma_{A2}^{2} \end{array}\right]\right)$ and $\left[\begin{array}{c} e_{1}\\ e_{2} \end{array}\right]\;\sim \;N\left(0,I⊗\left[\begin{array}{cc} \sigma_{e1}^{2} & \sigma_{e1e2}\\ \sigma_{e1e2} & \sigma_{e2}^{2} \end{array}\right]\right)$, where ***G*** is the matrix of additive genetic relationships constructed based on chip or iWGS data; ***I*** is the identity matrix; $\sigma_{A1}^{2}$ and $\sigma_{A2}^{2}$ are the additive genetic variance for NRs and CL, respectively; $\sigma_{e1}^{2}$ and $\sigma_{e2}^{2}$ are the residual variance for NRs and CL, respectively; $\sigma_{A1A2}$ is the additive genetic covariance between NRs and CL and $\sigma_{e1e2}$ is the residual covariance between NRs and CL. The GEBVs were defined as $\left[\begin{array}{cc} Z_{1} & 0\\ 0 & Z_{2} \end{array}\right]\left[\begin{array}{c} \hat{g_{1}}\\ \hat{g_{2}} \end{array}\right]$.

ST-MABLUP (the most significant SNPs identified by GWAS based on chip or iWGS data were added as a fixed effect to single-trait GBLUP):

y=Xa+Ms+Zg+e

where the meanings of y, a, g, e, X and Z are the same as in ST-GBLUP; s is the vector of fixed effects of the most significant SNPs identified by GWAS based on chip or iWGS data and M is the incidence matrix for the genotypes of the most significant SNPs. The GEBV was defined as $M\hat{s}+Z\hat{g}$.

MT-MABLUP (the most significant locus identified by GWAS based on chip or iWGS data was added as a fixed effect to multi-trait GBLUP):

$$\left[\begin{array}{c} y_{1}\\ y_{2} \end{array}\right]=\left[\begin{array}{cc} X_{1} & 0\\ 0 & X_{2} \end{array}\right]\left[\begin{array}{c} a_{1}\\ a_{2} \end{array}\right]+\left[\begin{array}{cc} M_{1} & 0\\ 0 & M_{2} \end{array}\right]\left[\begin{array}{c} s_{1}\\ s_{2} \end{array}\right]+\left[\begin{array}{cc} Z_{1} & 0\\ 0 & Z_{2} \end{array}\right]\left[\begin{array}{c} g_{1}\\ g_{2} \end{array}\right]+\left[\begin{array}{c} e_{1}\\ e_{2} \end{array}\right]$$

where the meanings of $y_{1}$, $y_{2}$, $a_{1}$, $a_{2}$, $g_{1}$, $g_{2}$, $e_{1}$, $e_{2}$, $X_{1}$, $X_{2}$, $Z_{1}$ and $Z_{2}$ are the same as in MT-GBLUP; $s_{1}$ and $s_{2}$ are the vector of fixed effects of the most significant SNPs identified by GWAS based on chip or iWGS data and $M_{1}$ and $M_{2}$ are the incidence matrices for the genotypes of the most significant SNPs. The GEBVs were defined as $\left[\begin{array}{cc} M_{1} & 0\\ 0 & M_{2} \end{array}\right]\left[\begin{array}{c} \hat{s_{1}}\\ \hat{s_{2}} \end{array}\right]+\left[\begin{array}{cc} Z_{1} & 0\\ 0 & Z_{2} \end{array}\right]\left[\begin{array}{c} \hat{g_{1}}\\ \hat{g_{2}} \end{array}\right]$.

ST-OneBLUP (the significant SNPs identified by GWAS based on iWGS data were combined with chip data in single-trait GBLUP):

y=Xa+Wd+e

where the meanings of y, a, e and X are the same as in ST-GBLUP and d is the vector of random polygenic effects. It is assumed that d~*N* (0, ***G***$\sigma_{A}^{2}$), where ***G*** is the matrix of additive genetic relationships constructed based on chip data plus significant SNPs identified by GWAS based on iWGS data and $\sigma_{A}^{2}$ is the additive genetic variance. The GEBV was defined as $W\hat{d}$.

MT-OneBLUP (the significant SNPs identified by GWAS based on iWGS data were combined with chip data in multi-trait GBLUP):

$$\left[\begin{array}{c} y_{1}\\ y_{2} \end{array}\right]=\left[\begin{array}{cc} X_{1} & 0\\ 0 & X_{2} \end{array}\right]\left[\begin{array}{c} a_{1}\\ a_{2} \end{array}\right]+\left[\begin{array}{cc} W_{1} & 0\\ 0 & W_{2} \end{array}\right]\left[\begin{array}{c} d_{1}\\ d_{2} \end{array}\right]+\left[\begin{array}{c} e_{1}\\ e_{2} \end{array}\right]$$

where the meanings of $y_{1}$, $y_{2}$, $a_{1}$, $a_{2}$, $e_{1}$, $e_{2}$, $X_{1}$ and $X_{2}$ are the same as in MT-GBLUP and $d_{1}$ and $d_{2}$ are the vectors of random polygenic effects. It is assumed that $\left[\begin{array}{c} d_{1}\\ d_{2} \end{array}\right]\;\sim \;N\left(0,G⊗\left[\begin{array}{cc} \sigma_{A1}^{2} & \sigma_{A1A2}\\ \sigma_{A1A2} & \sigma_{A2}^{2} \end{array}\right]\right)$, where ***G*** is the matrix of additive genetic relationships constructed by chip data plus significant SNPs obtained from GWAS based on iWGS data for NRs and CL; $\sigma_{A1}^{2}$ and $\sigma_{A2}^{2}$ are the additive genetic variance for NRs and CL, respectively; $\sigma_{e1}^{2}$ and $\sigma_{e2}^{2}$ are the additive residual variance for NRs and CL, respectively; $\sigma_{A1A2}$ is the additive covariance between NRs and CL and $W_{1}$ and $W_{2}$ are incidence matrices for SNP genotypes. The GEBVs were defined as $\left[\begin{array}{cc} W_{1} & 0\\ 0 & W_{2} \end{array}\right]\left[\begin{array}{c} \hat{d_{1}}\\ \hat{d_{2}} \end{array}\right]$.

ST-MultiBLUP (the significant SNPs identified by GWAS based on iWGS data were used as the second component of the random additive effect added into single-trait GBLUP):

$$y=Xa+Z_{1}g_{1}+Z_{2}g_{2}+e$$

where the meanings of y, a, e and X are the same as in ST-GBLUP and $g_{1}$ and $g_{2}$ are the vectors of random polygenic effects. It is assumed that $g_{1}\;\sim \;N\left(0,\;G_{1}\sigma_{A1}^{2}\right)$ and $g_{2}\;\sim \;N\left(0,\;G_{2}\sigma_{A2}^{2}\right)$, where $G_{1}$ is the matrix of additive genetic relationships constructed by all the significant SNPs obtained from GWAS based on iWGS data; $G_{2}$ is the matrix of additive genetic relationships constructed by the SNPs of the chip or the rest of the iWGS data and $\sigma_{A1}^{2}$ and $\sigma_{A2}^{2}$ are the variance of the first and the second component of additive genetic effect, respectively. $Z_{1}$ and $Z_{2}$ are incidence matrices. The GEBV was defined as $Z_{1}\hat{g_{1}}+Z_{2}\hat{g_{2}}$.

MT-MultiBLUP (the significant SNPs identified by GWAS based on iWGS data were used as the second component of the random additive effect added into multi-trait GBLUP):

$$\left[\begin{array}{c} y_{1}\\ y_{2} \end{array}\right]=\left[\begin{array}{cc} X_{1} & 0\\ 0 & X_{2} \end{array}\right]\left[\begin{array}{c} a_{1}\\ a_{2} \end{array}\right]+\left[\begin{array}{cc} Z_{11} & 0\\ 0 & Z_{21} \end{array}\right]\left[\begin{array}{c} g_{11}\\ g_{21} \end{array}\right]+\left[\begin{array}{cc} Z_{12} & 0\\ 0 & Z_{22} \end{array}\right]\left[\begin{array}{c} g_{12}\\ g_{22} \end{array}\right]+\left[\begin{array}{c} e_{1}\\ e_{2} \end{array}\right]$$

where the meanings of $y_{1}$, $y_{2}$, $a_{1}$, $a_{2}$, $e_{1}$, $e_{2}$, $X_{1}$ and $X_{2}$ are the same as in MT-GBLUP; $g_{11}$ and $g_{12}$ are the vectors of random polygenic effect for NRs and $g_{21}$ and $g_{22}$ are the vectors of random polygenic effect for CL. It is assumed that $\left[\begin{array}{c} g_{11}\\ g_{21} \end{array}\right]\;\sim \;N\left(0,G_{1}⊗\left[\begin{array}{cc} \sigma_{A11}^{2} & \sigma_{A11A21}\\ \sigma_{A11A21} & \sigma_{A21}^{2} \end{array}\right]\right)$ and $\left[\begin{array}{c} g_{12}\\ g_{22} \end{array}\right]\;\sim \;N\left(0,G_{2}⊗\left[\begin{array}{cc} \sigma_{A12}^{2} & \sigma_{A12A22}\\ \sigma_{A12A22} & \sigma_{A22}^{2} \end{array}\right]\right)$, where $G_{1}$ is the matrix of additive genetic relationships constructed by all the significant SNPs obtained from GWAS of NRs and CL based on iWGS data; $G_{2}$ is the matrix of additive genetic relationships constructed by the SNPs of chip or the rest of iWGS data; $\sigma_{A11}^{2}$ and $\sigma_{A21}^{2}$ are the variance of the first component of additive genetic effect for NRs and CL, respectively; $\sigma_{A12}^{2}$ and $\sigma_{A22}^{2}$ are the variance of the second component of additive genetic effect for NRs and CL, respectively, and $\sigma_{A11A21}$ and $\sigma_{A12A22}$ are the covariance of the first and second component of additive genetic effect between the two traits. $Z_{11}$, $Z_{12}$, $Z_{21}$ and $Z_{22}$ are incidence matrices. The GEBVs were defined as $\left[\begin{array}{cc} Z_{11} & 0\\ 0 & Z_{21} \end{array}\right]\left[\begin{array}{c} \hat{g_{11}}\\ \hat{g_{21}} \end{array}\right]+\left[\begin{array}{cc} Z_{12} & 0\\ 0 & Z_{22} \end{array}\right]\left[\begin{array}{c} \hat{g_{12}}\\ \hat{g_{22}} \end{array}\right]$.

In the above BLUP alphabet methods, the GEBVs were predicted using Hiblup software (Version 1.1.0) https://www.hiblup.com (accessed on 20 April 2022) with default settings.

### 2.4. GP Models of Bayesian Alphabet Method

$$y=Xa+\sum_{i=1}^{m}z_{i}g_{i}+e$$

where the meanings of y, a and e are the same as in ST-GBLUP; *m* is the number of SNP markers; $z_{i}$ is the genotype vector of the *i*-th SNP and $g_{i}$ is the additive effect of the *i*-th SNP. It is assumed that $g_{i}\;\sim \;N\left(0,\sigma_{i}^{2}\right)$, where $\sigma_{i}^{2}$ is the additive effect variance of the *i*-th SNP effect. The Bayesian alphabet methods make different assumptions about the prior distribution of $g_{i}$ and let *π* denote the proportion of SNPs, which have null effect on the trait. The assumptions of the Bayesian alphabet methods used in this study are as follows:

BayesA:

$$g_{i}\;|\;\left(\pi =0,\;\sigma_{i}^{2}\right)\;\sim \;\{\begin{array}{c} \sigma_{i}^{2}=0;\\ N\left(0,\sigma_{i}^{2}\right),\sigma_{i}^{2}\;\sim \chi^{-2}\left(v,S\right); \end{array}\begin{array}{c} \pi \\ 1-\pi \end{array}$$

BayesB:

$$g_{i}\;|\;\left(\pi =0.95,\;\sigma_{i}^{2}\right)\;\sim \;\{\begin{array}{c} \sigma_{i}^{2}=0;\\ N\left(0,\sigma_{i}^{2}\right),\sigma_{i}^{2}\;\sim \chi^{-2}\left(v,S\right); \end{array}\begin{array}{c} \pi \\ 1-\pi \end{array}$$

BayesBpi:

$$g_{i}\;|\;\left(\pi \;\sim \;U\left(0,1\right),\;\sigma_{i}^{2}\right)=\{\begin{array}{c} \sigma_{i}^{2}=0;\\ \sim \;N\left(0,\sigma_{i}^{2}\right),\sigma_{i}^{2}\;\sim \chi^{-2}\left(v,S\right); \end{array}\begin{array}{c} \pi \\ 1-\pi \end{array}$$

BayesC:

$$g_{i}\;|\;\left(\pi =0.95,\;\sigma^{2}\right)\;\sim \;\{\begin{array}{c} \sigma^{2}=0;\\ N\left(0,\sigma^{2}\right),\;\sigma^{2}\sim \chi^{-2}\left(v,S\right); \end{array}\begin{array}{c} \pi \\ 1-\pi \end{array}$$

BayesCpi:

$$g_{i}\;|\;\left(\pi \;\sim \;U\left(0,1\right),\;\sigma^{2}\right)\;\sim \;\{\begin{array}{c} \sigma^{2}=0;\\ N\left(0,\sigma^{2}\right),\;\sigma^{2}\sim \chi^{-2}\left(v,S\right); \end{array}\begin{array}{c} \pi \\ 1-\pi \end{array}$$

BayesLasso:

$$g_{i}\;|\;\left(\pi =0,\;\sigma_{i}^{2}\right)\;\sim \;\{\begin{array}{c} \sigma_{i}^{2}=0;\\ N\left(0,\sigma_{i}^{2}\right),\;\sigma_{i}^{2}\sim E\left(\lambda^{2}/2\right); \end{array}\begin{array}{c} \pi \\ 1-\pi \end{array}$$

BayesR:

$$g_{i}\;|\;\left(\pi_{1}+\pi_{2}+\pi_{3}+\pi_{4}=1,\sigma_{g}^{2}\right)\;\sim \;\{\begin{array}{c} \sigma_{g}^{2}=0;\;\;\;\;\;\;\;\;\;\;\;\pi_{1}=0.95\\ N\left(0,10^{-4}\sigma_{g}^{2}\right);\pi_{2}=0.02\\ N\left(0,10^{-3}\sigma_{g}^{2}\right);\pi_{3}=0.02\\ N\left(0,10^{-2}\sigma_{g}^{2}\right);\pi_{4}=0.01 \end{array},\sigma_{g}^{2}\sim \chi^{-2}\left(v,S\right)$$

In the above Bayesian alphabet methods, v represents the degrees of freedom, S is scale parameter, λ is rate parameter, $\sigma^{2}$ is the addictive genetics variance explained by partial SNPs, $\sigma_{i}^{2}$ is the addictive genetics variance explained by the *i*-th SNP and $\sigma_{g}^{2}$ is the total additive genetic variance explained by all SNPs. The GEBV was defined as $\sum_{i=1}^{m}z_{i}\hat{g_{i}}$ and was predicted using the R package of hibayes (Version 1.1.0) https://github.com/YinLiLin/hibayes (accessed on 11 July 2022) with the MCMC (Markov Chain Monte Carlo) iterations set to 50,000, the burn-in iteration set to 25,000 and the sampling frequency set to 1. The initial values of v, S λ, $\sigma^{2}$, $\sigma_{i}^{2}$ and $\sigma_{g}^{2}$ used the default settings in hibayes.

### 2.5. SNPs Datasets for Different GP Models

In the different GP models, different datasets are used, which are chip data, chip data plus significant iWGS SNPs and iWGS data. To prevent the reuse of genetic markers in the chip data plus the significant iWGS SNPs dataset, the SNPs in the chip data are removed if they intersect with significant iWGS SNPs. Table 1 shows the datasets used by the GP models.

### 2.6. Evaluation of Predictive Performance of Models

Using self-written R scripts, 513 Suhuai pigs were randomly divided approximately into 10 equal subsets. One subset was used as the validation set in turn, and the remaining nine subsets were used as the training set. The prediction accuracy, bias of the model and runtime were calculated to evaluate the predictive ability of the GP model. The same random seed was set for different models to ensure that the training and validation sets used in each fold of cross-validation were consistent. The prediction accuracy of the model was defined as the correlation coefficient between the predicted GEBV and the corrected phenotype, and the prediction bias was defined as the regression coefficient of the corrected phenotype on the GEBV. The Tukey HSD test was used to perform multiple comparisons for prediction accuracy across different models. The *t*-test was used to determine whether the bias of the models deviated from 1. The corrected phenotype was the residual value of the phenotype after the fixed effects were corrected for using the lm() function in R. All the analyses were run on a DELL PowerEdge R840 server with CentOS 7.9. The server had 4 chips (24-core Intel(R) Xeon(R) Gold 6252 CPU @ 2.10 GHz) and 1 TB of running memory.

## 3. Results

### 3.1. Imputation Accuracy and Significant SNPs Identified by GWAS

The average accuracies of genotype imputation before and after quality control were 0.967 and 0.980, respectively (Figure S1). It was indicated that the iWGS data could be used for follow-up analysis. The significant SNPs were detected by GWAS using training data, and thus, the significant SNPs differed in different folds of cross-validation. The average number of significant SNPs identified by GWAS using iWGS data was approximately 11,568 and 360 for NRs and CL, respectively (Table S1). The average number of overlapping SNPs between chip data and significant iWGS SNPs was approximately 33 and 2 for NRs and CL, respectively (Table S2).

### 3.2. Predictive Ability of Different BLUP Alphabet Methods for NRs

**Figure 1:**
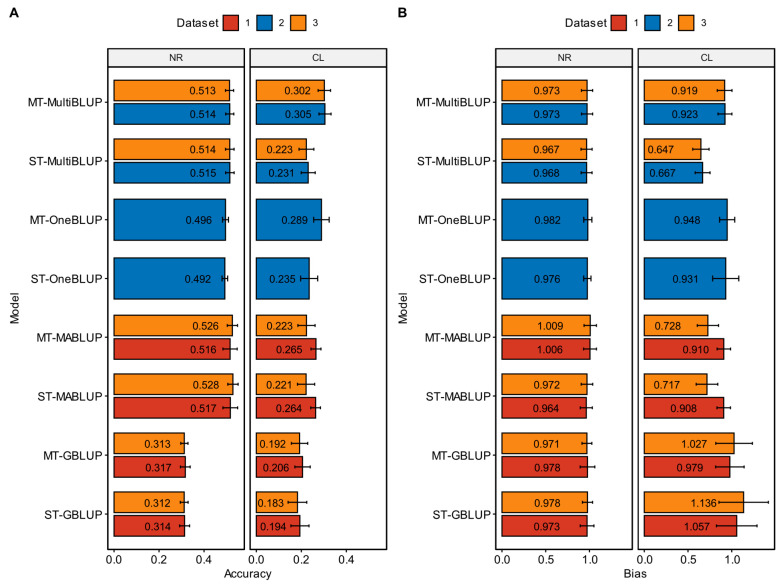
The accuracy (**A**) and bias (**B**) of different models of BLUP alphabet method for the number of ribs (NRs) and carcass length (CL). The *x*-axis shows the accuracy (**A**) or bias (**B**) and the *y*-axis indicates the genomic prediction models of BLUP alphabet method. The error line is standard error. Dataset 1 with red, 2 with blue and 3 with orange represent chip data, chip data plus significant whole-genome sequencing SNPs and imputation whole-genome sequencing data, respectively.

The left panel of Figure 1A and Figure 1B, respectively, shows the average accuracy and bias of prediction for NRs using BLUP alphabet methods based on different datasets. In the scenario of applying chip data and chip data plus significant iWGS SNPs, Tables S3 and S4 display the prediction accuracy and bias of different models in each fold of cross-validation, respectively. The prediction accuracy of ST-GBLUP was 0.314 ± 0.022, and the prediction accuracy of the MT-GBLUP was 0.317 ± 0.022. There was no significant difference in the prediction accuracy between ST-GBLUP and MT-GBLUP. The prediction accuracy of other BLUP alphabet methods, which integrated GWAS results, was significantly higher than that of ST-GBLUP and MT-GBLUP, and there was no significant difference in prediction accuracy among the BLUP alphabet methods integrating GWAS results. In the GP models of the BLUP alphabet method integrating GWAS results, the prediction accuracy of ST-OneBLUP was the lowest, with a prediction accuracy of 0.492 ± 0.013. The prediction accuracy of ST-MABLUP was the highest, with a prediction accuracy of 0.517 ± 0.033. The regression on prediction of all models did not significantly deviate from 1, indicating no significant bias.

In the scenario of applying iWGS data, Tables S5 and S6 showed the prediction accuracy and bias of different models in each cross-validation, respectively. The prediction accuracy of ST-GBLUP was 0.312 ± 0.017, and the prediction accuracy of MT-GBLUP was 0.313 ± 0.016. The difference in prediction accuracy between ST-GBLUP and MT-GBLUP was not significant. The prediction accuracy of the other BLUP alphabet methods, which integrated GWAS results, was significantly higher than that of ST-GBLUP and MT-GBLUP, and the difference in prediction accuracy among the BLUP alphabet methods that integrated GWAS results was not significant. Among the GP models of the BLUP alphabet method integrating GWAS results, the prediction accuracy of MT-MultiBLUP was the lowest, with a prediction accuracy of 0.513 ± 0.018. The prediction accuracy of ST-MABLUP was the highest, with a prediction accuracy of 0.528 ± 0.023. The regression on the prediction of all models did not significantly deviate from 1, indicating no significant bias.

### 3.3. Predictive Ability of Different Bayesian Alphabet Methods for NRs

**Figure 2:**
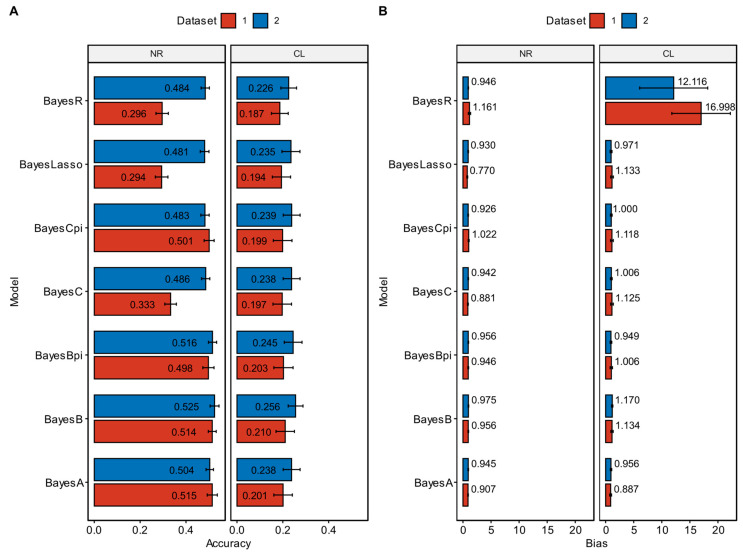
The accuracy (**A**) and bias (**B**) of different models of Bayesian alphabet method for the number of ribs (NRs) and carcass length (CL). The *x*-axis shows the accuracy (**A**) or bias (**B**) and the *y*-axis indicates the genomic prediction models of Bayesian alphabet method. The error line is standard error. Dataset 1 with red and 2 with blue represent chip data and chip data plus significant whole-genome sequencing SNPs, respectively.

The left panel of Figure 2A and Figure 2B, respectively, shows the average prediction accuracy and bias of the GP models of the Bayesian alphabet method for NRs. In the scenario of applying chip data, Tables S7 and S8 show the prediction accuracy and bias of different models in each fold of cross-validation, respectively. The BayesLasso had the lowest prediction accuracy, with a prediction accuracy of 0.294 ± 0.027, while the BayesA had the highest prediction accuracy, with a prediction accuracy of 0.515 ± 0.022. The differences in prediction accuracy among BayesA, BayesB, BayesBpi and BayesCpi were not significant, but the prediction accuracy of these four models was significantly higher than that of BayesC, BayesLasso and BayesR. The differences in prediction accuracy among BayesC, BayesLasso and BayesR were not significant. Except for BayesLasso, the regression on the prediction of other Bayesian alphabet methods did not significantly deviate from 1, indicating no significant bias.

In the scenario of applying chip data plus significant iWGS SNPs, Tables S9 and S10 show the prediction accuracy and bias of different models in each cross-validation fold, respectively. BayesLasso had the lowest prediction accuracy, with a prediction accuracy of 0.481 ± 0.019. BayesB had the highest prediction accuracy, with a prediction accuracy of 0.525 ± 0.019. Among Bayesian alphabet methods, the differences in prediction accuracy were not significant. The regression on the prediction of all Bayesian alphabet methods did not significantly deviate from 1, indicating no significant bias.

### 3.4. Predictive Ability of Different BLUP Alphabet Methods for CL

The right panel of Figure 1A and Figure 1B, respectively, shows the average accuracy and bias of the prediction for CL using BLUP alphabet methods based on different datasets. In the scenario of applying chip data and chip data plus significant iWGS SNPs, Tables S11 and S12 showed the prediction accuracy and bias of different models in each cross-validation, respectively. The prediction accuracy of ST-GBLUP was 0.194 ± 0.040, and the prediction accuracy of MT-GBLUP was 0.205 ± 0.034. There was no significant difference in prediction accuracy between ST-GBLUP and MT-GBLUP. In the GP models of the BLUP alphabet method integrating GWAS results, the prediction accuracy of ST-MultiBLUP was the lowest, with a prediction accuracy of 0.231 ± 0.031. The prediction accuracy of MT-MultiBLUP was the highest, with a prediction accuracy of 0.305 ± 0.027. There was no significant difference in prediction accuracy among all BLUP alphabet methods. Except for the regression on the prediction of ST-MultiBLUP, which significantly deviated from 1, the regression on the prediction of the other models did not significantly deviate from 1, indicating no significant bias.

In the scenario of applying iWGS data, Tables S13 and S14 show the prediction accuracy and bias of different models in each fold of cross-validation, respectively. The prediction accuracy of ST-GBLUP was 0.183 ± 0.042, and the prediction accuracy of MT-GBLUP was 0.192 ± 0.036. The difference in prediction accuracy between ST-GBLUP and MT-GBLUP was not significant. In the GP models of the BLUP alphabet method integrating GWAS results, the prediction accuracy of ST-MABLUP was the lowest, with a prediction accuracy of 0.221 ± 0.038. The prediction accuracy of MT-MultiBLUP was the highest, with a prediction accuracy of 0.302 ± 0.028. The differences in prediction accuracy among the BLUP alphabet methods were not significant. Except for the regression on the prediction of ST-MABLUP and ST-MultiBLUP, which significantly deviated from 1, the regression on the prediction of the other models did not significantly deviate from 1, indicating no significant bias.

### 3.5. Predictive Ability of Different Bayesian Alphabet Methods for CL

The right panel of Figure 2A and 2B, respectively, show the average prediction accuracy and bias of the GP models of the Bayesian alphabet method for CL. In the scenario of applying chip data, Tables S15 and S16 show the prediction accuracy and bias of different models in each fold cross-validation, respectively. BayesR had the lowest prediction accuracy, which was 0.187 ± 0.037, and BayesB had the highest prediction accuracy, which was 0.210 ± 0.040. There was no significant difference in the prediction accuracy among Bayesian alphabet methods. Except for BayesR, the regression on prediction of other Bayesian alphabet methods did not significantly deviate from 1, indicating no significant bias.

In the scenario of applying chip data plus significant iWGS SNPs, Tables S17 and S18 showed the prediction accuracy and bias of different models in each cross-validation fold, respectively. The prediction accuracy of the BayesR model was the lowest, with a prediction accuracy of 0.226 ± 0.034, while the prediction accuracy of BayesB was the highest, with a prediction accuracy of 0.256 ± 0.032. There was no significant difference in the prediction accuracy among Bayesian alphabet methods. Except for BayesR, the regression on the prediction of other Bayesian alphabet methods did not significantly deviate from 1, indicating no significant bias.

### 3.6. The Runtime of Different GP Models

The running time of GP models in cross-validation is displayed in Table 2. In the scenario of applying the BLUP alphabet method, ST-GBLUP had the shortest runtime, while MT-MultiBLUP had the longest runtime. The single-trait models had shorter runtimes than their corresponding multi-trait models. Within the same model, the model based on imputed data took longer to run than the model based on chip data or chip data plus significant iWGS SNPs. In the scenario of applying the Bayesian alphabet method, the BayesR model took the longest time, while the BayesC model took the shortest time. Within the same model, the model that only used chip data took less time than chip data plus significant iWGS SNPs.

## 4. Discussion

Although many models of GP have been proposed, no model has good predictive performance for all traits. This may be due to the fact that different traits have different genetic mechanisms, and the simple assumption of models cannot well reflect the complex genetic mechanisms. Therefore, the optimal GP models for different traits differ due to the complexity of trait genetic mechanisms and sample size. In this study, using three different datasets, the prediction accuracy, bias and running time of 15 different GP models for NRs and CL in the Suhuai pig population were evaluated by a 10-fold cross-validation.

Compared with re-sequencing or iWGS data, the SNP chip data are more convenient to obtain and have a lower price, making them more widely used in practical pig breeding. The use of re-sequencing or iWGS data can cover causal mutations or increase the probability of genetic markers highly linked to causal mutations that affect traits, thereby improving the prediction accuracy of GS models [26,27]. However, some studies have shown that the use of iWGS data can only slightly improve or not improve the prediction accuracy of GS [28,29]. This could be because the effects of the genetic markers affecting the trait are diluted by numerous ineffective genetic markers and imputation errors in iWGS data. However, this does not mean that re-sequencing or imputed data have no value in GS. Using GWAS to pre-select SNPs in re-sequencing or imputed data and their integration into the GP model have shown an improvement in prediction accuracy [30,31].

The loci with large effects identified by GWAS as fixed effects were incorporated into GBLUP, which could improve the prediction accuracy of the model [13]. In the current study, the most significant SNP identified by GWAS on the reference population was added into the GBLUP as a fixed effect (ST-MABLUP and MT-MABLUP). For NRs, the prediction accuracies of models ST-MABLUP and MT-MABLUP were significantly higher than ST-GBLUP and MT-GBLUP. Nevertheless, the prediction accuracy of ST-MABLUP and MT-MABLUP was not significantly different from the ST-GBLUP and MT-GBLUP for CL. This may be due to the effect size of the most significant SNP identified by GWAS for CL being small, and the estimation errors of SNP effects were large due to the limited sample size.

Previous studies have shown that, differentially, the SNPs associated with the trait and other SNPs could improve the predictive ability of the GP model. For instance, the SNPs associated with traits identified by GWAS based on iWGS data were incorporated into the GBLUP model as the second component additive genetic effect [30,32]. In this study, we added significant SNPs associated with traits identified by GWAS from iWGS data as the second component of additive genetic effect to single- and multi-trait GBLUP models. For NRs, both ST-MultiBLUP and MT-MultiBLUP were significantly higher than both ST-GBLUP and MT-GBLUP.

The multi-trait GBLUP model can improve the predictive ability by using the information of genetically correlated traits compared to the single-trait GBLUP model [18,19,33]. The advantage of the multi-trait model is large in the case that the target trait has a low heritability and small data of phenotypic records while the genetically correlated traits have high heritability and extensive phenotypic record data. In this study, the numbers of animals with records of NRs and CL in the reference population were the same. The heritability of NRs and CL in the experimental Suhuai pig population was 0.323 and 0.195, respectively, and the genetics correlation was 0.636 between NRs and CL [20]. However, compared to the single-trait model, the prediction accuracy of all corresponding multi-trait models did not significantly improve. This may be due to the limited sample size.

For NRs, only ST-MABLUP and MT-MABLUP based on iWGS data had higher prediction accuracy than those based on chip data in the GP models of the BLUP alphabet method. This may be because the most significant SNPs associated with NRs and identified by the imputed data GWAS were in stronger LD with causal mutations affecting NRs. The prediction accuracy of the rest of the BLUP alphabet methods based on iWGS data was lower than that of their corresponding chip-based GP models. A possible reason could be that many ineffective SNPs in the iWGS data act as noise, and/or there exists an imputation error.

Bayesian GP methods can improve accuracy when the trait is affected by genes with moderate effects or large effects. However, the Bayesian GP models had a limited ability to enhance accuracy when the trait was mainly affected by a large number of minor genes [34,35]. Previous research has shown that NRs were mainly influenced by several large-effect genes and CL was mainly influenced by polygene effects [20]. For NRs, this study found that the BayesA, BayesB, BayesBpi and BayesCpi models had significantly higher accuracy than the GBLUP model when using chip data. The prediction accuracy of the BayesA model improved by 20% compared with the GBLUP model.

Due to the sequential nature of the MCMC iterations in the Bayesian GP models, parallel computation was difficult. When chip data were imputed to the re-sequencing density, the marker density was increased by approximately 247-fold, resulting in a significant increase in computational time. Therefore, in this study, we merged the chip data and significant WGS SNPs to reduce the running time of the models and used this dataset to compare the predictive abilities of different Bayesian GP models. For NRs, using chip plus significant iWGS SNPs, there was no significant difference among seven Bayesian GP models, OneBLUP and MultiBLUP. Compared to using chip data alone, the inclusion of significant SNPs did not significantly improve the prediction accuracy in BayesA, BayesB, BayesBpi and BayesCpi models. On the other hand, the prediction accuracies of the BayesC, BayesLasso and BayesR models were significantly improved, possibly due to the enhanced compatibility between the effects of SNP markers and the assumptions of the models after incorporating the significant SNPs from the imputed GWAS data. For CL, using chip plus significant iWGS SNPs, there was no significant difference among seven Bayesian GP models, OneBLUP and MultiBLUP. Compared to using chip data alone, the inclusion of significant SNPs did not significantly improve the prediction accuracy in the seven Bayesian GP models. The prediction bias of the BayesR model also showed a significant deviation from 1, and the possible reason was that the effect of the genetic marker did not match the assumptions of the BayesR model for CL.

Based on the criteria of highest prediction accuracy, unbiased prediction and shortest running time, the optimal genomic selection models for NRs and CL were determined in the Suhuai pig population. For the genetic improvement of NRs, the optimal genome prediction model was based on the imputation data and treating the most significant SNP identified by GWAS as fixed effects in the GBLUP model; for the genetic improvement of CL, the optimal genome selection model was the multi-trait GBLUP using chip data plus the significant iWGS SNPs and treating the significant iWGS SNPs as the second component of additive genetic effect.

## 5. Conclusions

In the Suhuai pig population, for the genetic improvement of NRs, the optimal genomic prediction model was based on the imputation data and treating the most significant SNPs identified by GWAS as fixed effects into the GBLUP model; for the genetic improvement of CL, the optimal genomic prediction model was the multi-trait GBLUP using chip data plus the significant iWGS SNPs and treating the significant iWGS SNPs as the second component of additive genetic effect.

## Figures and Tables

**Table 1:** The dataset for different genomic prediction models.

Models	Dataset ^1^		
	1	2	3
ST-GBLUP	√	-	√
MT-GBLUP	√	-	√
ST-MABLUP	√	-	√
MT-MABLUP	√	-	√
ST-OneBLUP	-	√	-
MT-OneBLUP	-	√	-
ST-MultiBLUP	-	√	√
MT-MultiBLUP	-	√	√
BayesA	√	√	-
BayesB	√	√	-
BayesBpi	√	√	-
BayesC	√	√	-
BayesCpi	√	√	-
BayesLasso	√	√	-
BayesR	√	√	-

^1^ Datasets 1, 2 and 3 represent chip data, chip data plus significant whole-genome sequencing SNPs and imputation whole-genome sequencing data, respectively, the same as below. The symbol “√” indicates that the corresponding dataset is used.

**Table 2:** Computational time of genomic prediction using different models for the number of ribs and carcass length trait.

Model	Number of Ribs			Carcass Length		
	Dataset 1	Dataset 2	Dataset 3	Dataset 1	Dataset 2	Dataset 3
ST-GBLUP	16 s	-	1 min 42 s	17 s	-	1 min 45 s
MT-GBLUP	28 s	-	1 min 55 s	28 s	-	1 min 55 s
ST-MABLUP	3 min 43 s	-	6 h 57 min 33 s	3 min 51 s	-	6 h 57 min 33 s
MT-MABLUP	6 min 56 s	-	12 h 44 min 21 s	6 min 56 s	-	12 h 44 min 21 s
ST-OneBLUP	-	6 h 38 min 57 s	-	-	6 h 33 min 33 s	-
MT-OneBLUP	-	12 h 29 min 21 s	-	-	12 h 29 min 21 s	-
ST-MultiBLUP	-	6 h 59 min 21 s	7 h 2 min 21 s	-	6 h 32 min 57 s	6 h 49 min 9 s
MT-MultiBLUP	-	12 h 39 min 33 s	12 h 46 min 45 s	-	12 h 39 min 33 s	12 h 46 min 45 s
BayesA	10 h 33 min 22 s	19 h 22 min	-	10 h 26 min 46 s	16 h 48 min 55 s	-
BayesB	7 h 47 min 20 s	16 h 11 min 26 s	-	7 h 47 min 3 s	14 h 9 min 31 s	-
BayesBpi	7 h 51 min 31 s	16 h 14 min	-	7 h 53 min 46 s	14 h 16 min 30 s	-
BayesC	6 h 54 min 20 s	15 h 1 min 53 s	-	6 h 55 min 47 s	13 h 23 min 30 s	-
BayesCpi	7 h 6 min 32 s	15 h 51 min 56 s	-	7 h 24 min 10 s	13 h 55 min 23 s	-
BayesLasso	10 h 16 min 24 s	18 h 59 min 41 s	-	10 h 14 min 35 s	16 h 39 min 55 s	-
BayesR	11 h 5 min 1 s	20 h 13 min 11 s	-	10 h 51 min 6 s	17 h 19 min 47 s	-

## Data Availability

The script, phenotype and genotype data used in the present study are deposited in the fig share repository (https://doi.org/10.6084/m9.figshare.25244527) (accessed on 26 January 2025).

## Supplementary Materials

The following supporting information can be downloaded at https://www.mdpi.com/article/10.3390/ani15030412/s1, Table S1: The number of significant SNPs identified by GWAS using imputed whole-genome sequencing data in each fold of cross-validation. Table S2: The number of overlapping SNPs between chip data and significant imputed whole-genome sequencing SNPs in each fold of cross-validation. Table S3: The accuracy of different GP models of BLUP alphabet method based on chip data and chip data plus significant imputed whole-genome sequencing SNPs in each fold of cross-validation for NRs. Table S4: The bias of different GP models of BLUP alphabet method based on chip data and chip data plus significant imputed whole-genome sequencing SNPs in each fold of cross-validation for NRs trait. Table S5: The accuracy of different GP models of BLUP alphabet method based on imputation data in each fold of cross-validation for NRs trait. Table S6: The bias of different GP models of BLUP alphabet method based on imputation data in each fold of cross-validation for NRs trait. Table S7: The accuracy of different GP models of Bayesian alphabet method based on chip data in each fold of cross-validation for NRs trait. Table S8: The bias of different GP models of Bayesian alphabet method based on chip data in each fold of cross-validation for NRs trait. Table S9: The accuracy of different GP models of Bayesian alphabet method in each fold of cross-validation based on chip data plus significant imputed whole-genome sequencing SNPs for NRs trait. Table S10: The bias of different GP models of Bayesian alphabet method in each fold of cross-validation based on chip data plus significant imputed whole-genome sequencing SNPs for NRs trait. Table S11: The accuracy of different GP models of BLUP alphabet method based on chip data and chip data plus significant imputed whole-genome sequencing SNPs in each fold of cross-validation for CL trait. Table S12: The bias of different GP models of BLUP alphabet method based on chip data and chip data plus significant imputed whole-genome sequencing SNPs in each fold of cross-validation for CL trait. Table S13: The accuracy of different GP models of BLUP alphabet method based on imputation data in each fold of cross-validation for CL trait. Table S14: The bias of different GP models of BLUP alphabet method based on imputation data in each fold of cross-validation for CL trait. Table S15: The accuracy of different GP models of Bayesian alphabet method based on chip data in each fold of cross-validation for CL trait. Table S16: The bias of different GP models of Bayesian alphabet method based on chip data in each fold of cross-validation for CL trait. Table S17: The accuracy of different GP models of Bayesian alphabet method in each fold of cross-validation based on chip data plus significant imputed whole-genome sequencing SNPs for CL trait. Table S18: The bias of different GP models of Bayesian alphabet method in each fold of cross-validation based on chip data plus significant imputed whole-genome sequencing SNPs for CL trait. Figure S1: Accuracy of imputation to whole-genome sequence by chromosome.
