# Supplementary material for: Integrating Significant SNPs Identified by GWAS for Genomic Prediction of the Number of Ribs and Carcass Length in Suhuai Pigs
Source: Animals (Basel). 2025 Feb 2;15(3):412. doi: 10.3390/ani15030412 (PMC11816145; doi:10.3390/ani15030412)

Table S1 The number of significant SNPs identified by GWAS using imputed whole-genome sequencing data in each fold of cross validation for the number of ribs (NR) and carcass length (CL) traits.

	Fold										
**Trait**	**1**	**2**	**3**	**4**	**5**	**6**	**7**	**8**	**9**	**10**	**Mean**
NR	11,479	11,717	12,215	11,441	11,678	11,307	10,609	13,161	10,688	11,379	11,567.4
CL	359	418	144	150	534	256	334	535	359	508	359.7

Table S2 The number of overlapping SNPs between chip data and significant imputed whole-genome sequencing SNPs in each fold of cross validation for the number of ribs (NR) and carcass length (CL) traits.

	Fold										
**Trait**	**1**	**2**	**3**	**4**	**5**	**6**	**7**	**8**	**9**	**10**	**Mean**
Number of ribs	37	32	33	28	33	37	34	36	30	29	32.9
Carcass length	1	5	1	1	2	1	1	1	1	2	1.6

Table S3 The accuracy of different genomic prediction models of BLUP alphabet method based on chip data and chip data plus significant imputed whole-genome sequencing SNPs in each fold of cross validation for the number of ribs trait.

	**Fold**										
**Model**	**1**	**2**	**3**	**4**	**5**	**6**	**7**	**8**	**9**	**10**	**Mean ± SE**
ST-GBLUP	0.341	0.399	0.403	0.304	0.215	0.193	0.276	0.348	0.307	0.349	0.314 ± 0.022
MT-GBLUP	0.333	0.444	0.360	0.297	0.221	0.211	0.287	0.357	0.318	0.339	0.317 ± 0.022
ST-MABLUP	0.602	0.573	0.478	0.449	0.503	0.571	0.462	0.302	0.549	0.678	0.517 ± 0.033
MT-MABLUP	0.601	0.578	0.488	0.447	0.484	0.575	0.461	0.309	0.549	0.665	0.516 ± 0.032
ST-OneBLUP	0.560	0.526	0.448	0.475	0.456	0.484	0.489	0.454	0.485	0.548	0.492 ± 0.013
MT-OneBLUP	0.558	0.548	0.439	0.476	0.455	0.508	0.497	0.452	0.487	0.535	0.495 ± 0.013
ST-MultiBLUP	0.589	0.565	0.450	0.487	0.448	0.558	0.434	0.500	0.524	0.592	0.515 ± 0.019
MT-MultiBLUP	0.580	0.574	0.450	0.500	0.450	0.574	0.448	0.486	0.502	0.579	0.514 ± 0.018

Table S4 The bias of different genomic prediction models of BLUP alphabet method based on chip data and chip data plus significant imputed whole-genome sequencing SNPs in each fold of cross validation for the number of ribs trait.

	**Fold**										
**Model**	**1**	**2**	**3**	**4**	**5**	**6**	**7**	**8**	**9**	**10**	**Mean ± SE**
ST-GBLUP	1.030	1.378	1.236	0.883	0.607	0.624	0.813	0.987	1.052	1.120	0.973 ± 0.101
MT-GBLUP	0.979	1.555	1.065	0.852	0.626	0.672	0.808	1.030	1.085	1.103	0.978 ± 0.105
ST-MABLUP	1.164	1.082	0.913	0.662	0.778	1.244	0.769	0.733	1.088	1.205	0.964 ± 0.071
MT-MABLUP	1.209	1.139	0.984	0.685	0.774	1.322	0.792	0.778	1.146	1.236	1.006 ± 0.076
ST-OneBLUP	1.175	1.012	0.909	0.786	0.820	1.138	0.893	0.920	1.021	1.086	0.976 ± 0.051
MT-OneBLUP	1.188	1.060	0.909	0.785	0.811	1.207	0.879	0.917	1.001	1.058	0.982 ± 0.054
ST-MultiBLUP	1.208	1.045	0.864	0.739	0.715	1.243	0.722	1.005	1.072	1.062	0.968 ± 0.067
MT-MultiBLUP	1.205	1.084	0.874	0.763	0.730	1.316	0.737	0.962	1.025	1.035	0.973 ± 0.067

Table S5 The accuracy of different genomic prediction models of BLUP alphabet method based on imputation data in each fold of cross validation for the number of ribs trait.

	**Fold**										
**Model**	**1**	**2**	**3**	**4**	**5**	**6**	**7**	**8**	**9**	**10**	**Mean ± SE**
ST-GBLUP	0.327	0.355	0.406	0.336	0.234	0.225	0.284	0.326	0.294	0.331	0.312 ± 0.017
MT-GBLUP	0.328	0.390	0.371	0.325	0.234	0.235	0.290	0.330	0.300	0.323	0.313 ± 0.016
ST-MABLUP	0.602	0.561	0.474	0.450	0.504	0.582	0.446	0.467	0.538	0.661	0.528 ± 0.023
MT-MABLUP	0.601	0.562	0.483	0.449	0.491	0.585	0.442	0.460	0.536	0.651	0.526 ± 0.023
ST-MultiBLUP	0.592	0.554	0.447	0.489	0.450	0.564	0.433	0.499	0.523	0.593	0.514 ± 0.019
MT-MultiBLUP	0.582	0.562	0.447	0.504	0.452	0.578	0.445	0.485	0.499	0.579	0.513 ± 0.018

Table S6 The bias of different genomic prediction models of BLUP alphabet method based on imputation data in each fold of cross validation for the number of ribs trait.

	Fold										
**Model**	**1**	**2**	**3**	**4**	**5**	**6**	**7**	**8**	**9**	**10**	**Mean ± SE**
ST-GBLUP	0.988	1.172	1.274	1.065	0.656	0.768	0.870	0.923	1.018	1.043	0.978 ± 0.058
MT-GBLUP	0.962	1.289	1.132	1.006	0.648	0.800	0.855	0.948	1.040	1.033	0.971 ± 0.056
ST-MABLUP	1.173	1.047	0.904	0.666	0.782	1.270	0.725	0.908	1.044	1.198	0.972 ± 0.066
MT-MABLUP	1.208	1.095	0.966	0.689	0.789	1.351	0.739	0.933	1.089	1.231	1.009 ± 0.071
ST-MultiBLUP	1.221	1.016	0.862	0.744	0.716	1.260	0.715	1.002	1.073	1.062	0.967 ± 0.063
MT-MultiBLUP	1.214	1.054	0.871	0.769	0.730	1.326	0.725	0.978	1.025	1.034	0.973 ± 0.064

Table S7 The accuracy of different genomic prediction models of Bayesian alphabet method based on chip data in each fold of cross validation for the number of ribs trait.

	**Fold**										
**Model**	**1**	**2**	**3**	**4**	**5**	**6**	**7**	**8**	**9**	**10**	**Mean ± SE**
BayesA	0.588	0.552	0.463	0.474	0.446	0.528	0.531	0.420	0.495	0.649	0.515 ± 0.022
BayesB	0.597	0.561	0.479	0.481	0.463	0.551	0.520	0.415	0.530	0.548	0.514 ± 0.017
BayesBpi	0.634	0.515	0.482	0.481	0.401	0.555	0.540	0.383	0.438	0.550	0.498 ± 0.024
BayesC	0.391	0.427	0.379	0.346	0.161	0.252	0.354	0.320	0.297	0.402	0.333 ± 0.025
BayesCpi	0.503	0.405	0.496	0.531	0.434	0.584	0.504	0.404	0.549	0.600	0.501 ± 0.022
BayesLasso	0.352	0.399	0.350	0.307	0.105	0.201	0.311	0.298	0.258	0.358	0.294 ± 0.027
BayesR	0.343	0.409	0.369	0.304	0.118	0.209	0.310	0.295	0.250	0.354	0.296 ± 0.027

Table S8 The bias of different genomic prediction models of Bayesian alphabet method based on chip data in each fold of cross validation for the number of ribs trait.

Model	Fold										Mean ± SE
	**1**	**2**	**3**	**4**	**5**	**6**	**7**	**8**	**9**	**10**	
BayesA	1.096	0.945	0.834	0.697	0.675	1.164	0.898	0.701	0.928	1.135	0.907 ± 0.058
BayesB	1.148	0.998	0.899	0.722	0.719	1.246	0.938	0.750	1.104	1.035	0.956 ± 0.058
BayesBpi	1.303	0.952	0.917	0.725	0.644	1.304	0.962	0.693	0.915	1.044	0.946 ± 0.072
BayesC	1.076	1.158	0.943	0.822	0.348	0.764	0.946	0.734	0.880	1.142	0.881 ± 0.076
BayesCpi	1.354	1.155	0.939	0.788	0.638	1.392	1.118	0.695	1.176	0.960	1.022 ± 0.083
BayesLasso	0.945	1.113	0.854	0.713	0.217	0.585	0.824	0.672	0.758	1.024	0.770 ± 0.080
BayesR	1.078	2.456	1.054	0.802	0.270	0.703	1.887	0.751	1.467	1.140	1.161 ± 0.200

Table S9 The accuracy of different genomic prediction models of Bayesian alphabet method in each fold of cross validation based on chip data plus significant imputed whole-genome sequencing SNPs for the number of ribs trait.

	**Fold**										
**Model**	**1**	**2**	**3**	**4**	**5**	**6**	**7**	**8**	**9**	**10**	**Mean ± SE**
BayesA	0.584	0.546	0.464	0.492	0.429	0.521	0.520	0.438	0.484	0.559	0.504 ± 0.016
BayesB	0.599	0.566	0.477	0.492	0.456	0.556	0.517	0.445	0.509	0.629	0.525 ± 0.019
BayesBpi	0.596	0.559	0.469	0.491	0.444	0.544	0.517	0.451	0.490	0.597	0.516 ± 0.018
BayesC	0.566	0.529	0.443	0.492	0.393	0.495	0.521	0.419	0.455	0.546	0.486 ± 0.018
BayesCpi	0.564	0.528	0.441	0.491	0.389	0.484	0.519	0.418	0.449	0.546	0.483 ± 0.018
BayesLasso	0.564	0.525	0.430	0.490	0.379	0.488	0.524	0.418	0.451	0.544	0.481 ± 0.019
BayesR	0.563	0.527	0.447	0.491	0.390	0.486	0.523	0.416	0.454	0.543	0.484 ± 0.018

Table S10 The bias of different genomic prediction models of Bayesian alphabet method in each fold of cross validation based on chip data plus significant imputed whole-genome sequencing SNPs for the number of ribs trait.

	**Fold**										
**Model**	**1**	**2**	**3**	**4**	**5**	**6**	**7**	**8**	**9**	**10**	**Mean ± SE**
BayesA	1.174	0.986	0.872	0.748	0.694	1.227	0.898	0.790	0.963	1.099	0.945 ± 0.057
BayesB	1.170	1.023	0.906	0.743	0.728	1.284	0.894	0.804	1.033	1.164	0.975 ± 0.061
BayesBpi	1.169	0.999	0.883	0.742	0.714	1.254	0.899	0.813	0.969	1.118	0.956 ± 0.057
BayesC	1.171	0.970	0.870	0.808	0.660	1.184	0.966	0.776	0.950	1.067	0.942 ± 0.054
BayesCpi	1.153	0.963	0.864	0.795	0.653	1.137	0.949	0.768	0.925	1.058	0.926 ± 0.051
BayesLasso	1.173	0.959	0.811	0.806	0.617	1.163	1.006	0.779	0.939	1.052	0.930 ± 0.056
BayesR	1.158	0.986	0.904	0.817	0.657	1.142	0.993	0.772	0.963	1.068	0.946 ± 0.051

Table S11 The accuracy of different genomic prediction models of BLUP alphabet method based on chip data and chip data plus significant imputed whole-genome sequencing SNPs in each fold of cross validation for the carcass length trait.

	**Fold**										
**Model**	**1**	**2**	**3**	**4**	**5**	**6**	**7**	**8**	**9**	**10**	**Mean ± SE**
ST-GBLUP	0.161	-0.003	0.031	0.149	0.148	0.205	0.383	0.361	0.207	0.298	0.194 ± 0.040
MT-GBLUP	0.136	0.016	0.094	0.237	0.126	0.228	0.361	0.286	0.285	0.286	0.205 ± 0.034
ST-MABLUP	0.221	0.219	0.257	0.418	0.175	0.250	0.324	0.288	0.221	0.262	0.264 ± 0.022
MT-MABLUP	0.223	0.222	0.251	0.427	0.169	0.256	0.322	0.283	0.229	0.270	0.265 ± 0.022
ST-OneBLUP	0.205	0.078	0.056	0.232	0.152	0.250	0.402	0.416	0.271	0.287	0.235 ± 0.038
MT-OneBLUP	0.214	0.110	0.175	0.340	0.261	0.262	0.474	0.318	0.413	0.327	0.289 ± 0.034
ST-MultiBLUP	0.161	-0.003	0.031	0.149	0.148	0.205	0.383	0.361	0.207	0.298	0.231 ± 0.031
MT-MultiBLUP	0.136	0.016	0.094	0.237	0.126	0.228	0.361	0.286	0.285	0.286	0.305 ± 0.027

Table S12 The bias of different genomic prediction models of BLUP alphabet method based on chip data and chip data plus significant imputed whole-genome sequencing SNPs in each fold of cross validation for the carcass length trait.

	Fold										
**Model**	**1**	**2**	**3**	**4**	**5**	**6**	**7**	**8**	**9**	**10**	**Mean ± SE**
ST-GBLUP	1.006	-0.020	0.116	0.760	0.783	1.165	2.144	2.227	1.070	1.322	1.057 ± 0.232
MT-GBLUP	0.676	0.109	0.330	1.134	0.658	1.170	1.724	1.428	1.363	1.194	0.979 ± 0.163
ST-MABLUP	0.728	1.201	0.935	1.383	0.612	0.798	1.007	0.986	0.710	0.723	0.908 ± 0.077
MT-MABLUP	0.760	1.231	0.885	1.397	0.588	0.833	1.007	0.881	0.749	0.766	0.910 ± 0.077
ST-OneBLUP	0.891	0.394	0.188	1.039	0.563	1.096	1.516	1.731	1.013	0.883	0.931 ± 0.149
MT-OneBLUP	0.836	0.509	0.600	1.132	0.911	1.010	1.433	1.008	1.193	0.846	0.948 ± 0.087
ST-MultiBLUP	1.006	-0.020	0.116	0.760	0.783	1.165	2.144	2.227	1.070	1.322	0.667 ± 0.085
MT-MultiBLUP	0.676	0.109	0.330	1.134	0.658	1.170	1.724	1.428	1.363	1.194	0.923 ± 0.079

Table S13 The accuracy of different genomic prediction models of BLUP alphabet method based on imputation data in each fold of cross validation for the carcass length trait.

	Fold										
**Model**	**1**	**2**	**3**	**4**	**5**	**6**	**7**	**8**	**9**	**10**	**Mean ± SE**
ST-GBLUP	0.138	-0.005	-0.005	0.143	0.153	0.214	0.418	0.316	0.202	0.255	0.183 ± 0.042
MT-GBLUP	0.105	0.006	0.062	0.228	0.120	0.266	0.379	0.237	0.289	0.231	0.192 ± 0.036
ST-MABLUP	0.213	-0.004	0.188	0.422	0.164	0.271	0.376	0.249	0.203	0.128	0.221 ± 0.038
MT-MABLUP	0.209	0.001	0.188	0.427	0.154	0.279	0.373	0.256	0.206	0.138	0.223 ± 0.038
ST-MultiBLUP	0.230	0.214	-0.026	0.306	0.161	0.288	0.303	0.332	0.241	0.180	0.223 ± 0.033
MT-MultiBLUP	0.246	0.278	0.203	0.415	0.302	0.266	0.464	0.226	0.378	0.246	0.302 ± 0.028

Table S14 The bias of different genomic prediction models of BLUP alphabet method based on imputation data in each fold of cross validation for the carcass length trait.

	Fold										
**Model**	**1**	**2**	**3**	**4**	**5**	**6**	**7**	**8**	**9**	**10**	**Mean ± SE**
ST-GBLUP	0.881	-0.039	-0.021	0.747	0.919	1.409	2.934	2.068	1.207	1.256	1.136 ± 0.282
MT-GBLUP	0.538	0.044	0.229	1.147	0.673	1.602	2.129	1.232	1.634	1.041	1.027 ± 0.209
ST-MABLUP	0.718	-0.018	0.638	1.359	0.592	0.830	1.205	0.838	0.651	0.358	0.717 ± 0.124
MT-MABLUP	0.727	0.007	0.631	1.356	0.553	0.869	1.202	0.857	0.677	0.396	0.728 ± 0.121
ST-MultiBLUP	0.790	0.838	-0.093	0.766	0.512	0.805	0.843	0.899	0.612	0.503	0.647 ± 0.093
MT-MultiBLUP	0.861	1.153	0.712	1.214	1.007	0.922	1.225	0.497	1.066	0.533	0.919 ± 0.084

Table S15 The accuracy of different genomic prediction models of Bayesian alphabet method based on chip data in each fold of cross validation for the carcass length trait.

	**Fold**										
**Model**	**1**	**2**	**3**	**4**	**5**	**6**	**7**	**8**	**9**	**10**	**Mean ± SE**
BayesA	0.166	0.002	0.047	0.147	0.142	0.208	0.372	0.394	0.241	0.291	0.201 ± 0.040
BayesB	0.166	-0.008	0.078	0.160	0.174	0.222	0.396	0.380	0.227	0.310	0.210 ± 0.040
BayesBpi	0.173	0.007	0.013	0.163	0.146	0.220	0.391	0.384	0.236	0.295	0.203 ± 0.042
BayesC	0.164	-0.004	0.041	0.153	0.139	0.209	0.383	0.372	0.218	0.299	0.197 ± 0.040
BayesCpi	0.161	-0.002	0.033	0.153	0.153	0.209	0.383	0.377	0.228	0.296	0.199 ± 0.041
BayesLasso	0.153	-0.013	0.049	0.150	0.157	0.205	0.380	0.357	0.199	0.303	0.194 ± 0.040
BayesR	0.132	-0.038	0.131	0.140	0.179	0.224	0.384	0.272	0.147	0.298	0.187 ± 0.037

Table S16 The bias of different genomic prediction models of Bayesian alphabet method based on chip data in each fold of cross validation for the carcass length trait.

	Fold										
**Model**	**1**	**2**	**3**	**4**	**5**	**6**	**7**	**8**	**9**	**10**	**Mean ± SE**
BayesA	0.914	0.011	0.199	0.679	0.715	1.046	1.535	1.734	0.964	1.076	0.887 ± 0.167
BayesB	1.113	-0.060	0.407	0.885	1.074	1.373	2.021	1.989	1.115	1.422	1.134 ± 0.202
BayesBpi	1.053	0.046	0.041	0.818	0.679	1.156	1.794	2.127	1.164	1.185	1.006 ± 0.210
BayesC	1.104	-0.026	0.184	0.839	0.767	1.284	2.246	2.282	1.157	1.414	1.125 ± 0.239
BayesCpi	1.005	-0.017	0.126	0.808	0.897	1.222	2.147	2.420	1.122	1.447	1.118 ± 0.243
BayesLasso	1.055	-0.099	0.208	0.840	0.941	1.297	2.226	2.287	1.126	1.453	1.133 ± 0.239
BayesR	8.851	-1.878	16.379	7.230	14.503	9.350	48.765	22.286	2.630	41.864	16.998 ± 5.212

Table S17 The accuracy of different genomic prediction models of Bayesian alphabet method in each fold of cross validation based on chip data plus significant imputed whole-genome sequencing SNPs for the carcass length trait.

	Fold										
**Model**	**1**	**2**	**3**	**4**	**5**	**6**	**7**	**8**	**9**	**10**	**Mean ± SE**
BayesA	0.203	0.066	0.089	0.238	0.152	0.250	0.400	0.415	0.280	0.290	0.238 ± 0.037
BayesB	0.208	0.095	0.146	0.279	0.175	0.278	0.410	0.399	0.267	0.299	0.256 ± 0.032
BayesBpi	0.210	0.107	0.033	0.273	0.158	0.276	0.407	0.419	0.277	0.290	0.245 ± 0.039
BayesC	0.200	0.082	0.070	0.243	0.154	0.258	0.403	0.411	0.273	0.288	0.238 ± 0.037
BayesCpi	0.208	0.077	0.072	0.237	0.165	0.255	0.402	0.413	0.273	0.284	0.239 ± 0.037
BayesLasso	0.207	0.081	0.029	0.227	0.156	0.257	0.402	0.412	0.287	0.296	0.235 ± 0.039
BayesR	0.178	0.048	0.162	0.226	0.185	0.272	0.430	0.326	0.139	0.290	0.226 ± 0.034

Table S18 The bias of different genomic prediction models of Bayesian alphabet method in each fold of cross validation based on chip data plus significant imputed whole-genome sequencing SNPs for the carcass length trait.

	**Fold**										
**Model**	**1**	**2**	**3**	**4**	**5**	**6**	**7**	**8**	**9**	**10**	**Mean ± SE**
BayesA	0.919	0.372	0.369	1.043	0.641	1.122	1.452	1.665	1.015	0.962	0.956 ± 0.132
BayesB	1.039	0.590	0.737	1.392	0.830	1.348	1.728	1.759	1.116	1.156	1.170 ± 0.125
BayesBpi	0.853	0.506	0.099	1.199	0.572	1.222	1.498	1.569	1.038	0.933	0.949 ± 0.145
BayesC	0.914	0.442	0.259	1.155	0.625	1.213	1.626	1.828	1.085	0.910	1.006 ± 0.155
BayesCpi	0.920	0.382	0.274	1.091	0.651	1.227	1.653	1.814	1.067	0.922	1.000 ± 0.156
BayesLasso	0.816	0.402	0.084	1.055	0.621	1.313	1.496	1.859	0.938	1.130	0.971 ± 0.166
BayesR	11.094	0.402	9.993	1.559	3.276	8.401	65.294	11.944	7.785	1.415	12.116 ± 6.062

Figure S1 Accuracy of imputation to whole-genome sequence by chromosome. The average accuracy of imputation shows in histogram before (light blue) and after (red) filtering.

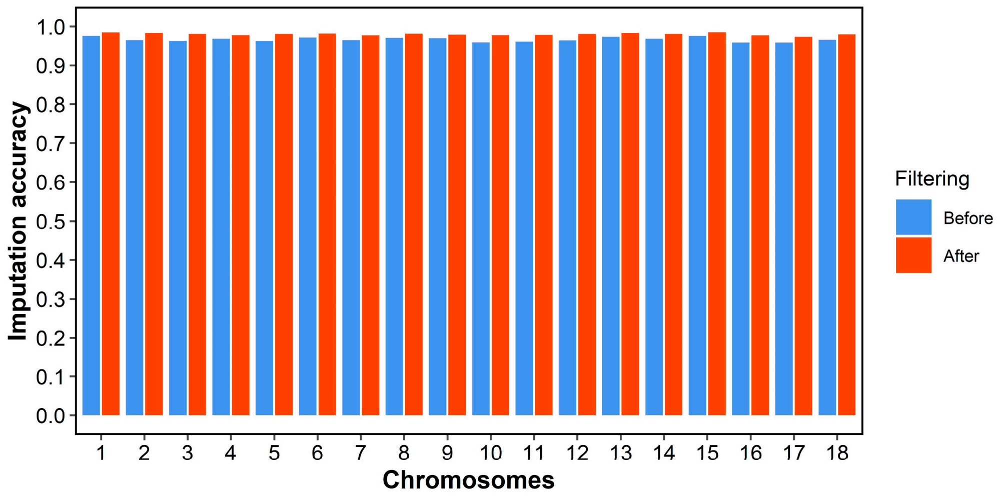

Supplement: Supplementary file 1 [file animals-15-00412-s001.zip › animals-3404200-supplementary.pdf]
